# QUALITY OF LIFE AFTER LAPAROSCOPIC SLEEVE GASTRECTOMY USİNG BAROS SYSTEM

**DOI:** 10.1590/0102-672020180001e1385

**Published:** 2018-08-16

**Authors:** Cüneyt KIRKIL, Erhan AYGEN, Mehmet Fatih KORKMAZ, Mehmet Buğra BOZAN

**Affiliations:** 1 Department of Surgery, Firat University Medical Faculty; 2 Department of Surgery, Turkish Ministry of Health Medical Sciences University, Elazig Training and Research Hospital, Elazig, Turkey.

**Keywords:** Obesity, Sleeve gastrectomy, Quality of life, Bariatric surgery, Obesidade, Gastrectomia vertical, Qualidade de vida, Cirurgia bariátrica

## Abstract

**Background:**

Laparoscopic sleeve gastrectomy (LSG) is currently the most frequently performed bariatric procedure in Turkey. The goal of weight reduction surgery is not only to decrease excess weight, but also to improve obesity related comorbidities and quality of life (QoL).

**Aim:**

To evaluate the impact of LSG on patient quality of life, weight loss, and comorbidities associated with morbid obesity according to the updated BAROS criteria.

**Methods:**

Eleven hundred thirty-eight adult patients were undergone to LSG by our bariatric surgery team between January 2013 and January 2016. A questionnaire (The Bariatric Analysis and Reporting Outcome System - BAROS) was published on social media. The data on postoperative complications were collected from hospital database.

**Results:**

Number of respondants was 562 (49.4%). Six of 1138 patients(0.5%) had leakage. All patients who had leakage were respondants. The overall complication rate was 7.7%. After a mean period of 7.4±5.3 months(1-30), mean excess weight loss was 71.3±27.1% (10.2-155.4). The respondants reported 772 comorbidities. Of these, 162 (30%) were improved, and 420 (54.4%) were resolved. The mean scores for QoL were significantly increased after LSG (range, p<0.05 to <0.001). Of the 562 patients, 26 (4.6%) were classified as failures; 86 (15.3%) fair; 196 (34.9%) good; 144 (25.6%) very good, and 110 (19.6%) excellent results according to the updated BAROS scoring system.

**Conclusion:**

LSG is a highly effective bariatric procedure in the manner of weight control, improvement in comorbidities and increasing of QoL in short- and mid-term.

## RESUMO

**Racional:**

A gastrectomia vertical laparoscópica (LSG) é atualmente o procedimento bariátrico mais frequentemente realizado na Turquia. O objetivo da operação de redução de peso não é apenas diminuir o excesso de peso, mas também melhorar as comorbidades e a qualidade de vida relacionadas à obesidade (QoL).

**Objetivo:**

Avaliar o impacto do LSG na qualidade de vida dos pacientes, perda de peso e comorbidades associadas à obesidade mórbida de acordo com os critérios BAROS atualizados.

**Métodos:**

Estudo não-randomizado de intervenção comportamental e de saúde pública. Um total de 1138 pacientes adultos foram submetidos a LSG entre janeiro de 2013 e janeiro de 2016. Um questionário (The Bariatric Analysis and Reporting Outcome System - BAROS foi utilizado. Os dados sobre complicações pós-operatórias foram coletados do banco de dados hospitalar.

**Resultados:**

Responderam ao questionário 562 (49,4%) pacientes. Seis de 1138 pacientes (0,5%) tiveram deiscência e todos estes responderam a pesquisa. A taxa geral de complicações foi de 7,7%. Após período médio de 7,4±5,3 meses (1-30), a perda média de excesso de peso foi de 71,3±27,1% (10,2-155,4). Os questionados relataram 772 comorbidades. Destes, 162 (30,0%) foram melhorados e 420 (54,4%) foram resolvidos.Os escores médios de QoL foram significativamente aumentados após LSG (p<0,05 a <0,001). Dentre os resultados dos 562 pacientes, 26 (4,6%) foram classificadas como falhas; 86 (15,3%) regular; 196 (34,9%) bom;144 (25,6%) muito bom; e 110 (19,6%) excelente de acordo com para o sistema de pontuação BAROS atualizado.

**Conclusão:**

O LSG é procedimento bariátrico altamente efetivo para controle de peso, melhora nas comorbidades e aumento da QoL em curto e meio prazos.

## INTRODUCTION

Obesity is a globally increasing health problem which impacts all age groups, races, and countries. It is a chronic disease associated with a variety of comorbidities such as diabetes, hypertension, coronary heart disease, and obstructive sleep apnea^6^ . Bariatric surgery is currently considered the most effective treatment option for morbid obesity. Laparoscopic sleeve gastrectomy (LSG) is currently the most frequently performed procedure in Turkey, USA/Canada and the Asia/Pacific regions alike^1^. 

The goal of weight reduction surgery is not only to decrease excess weight, but also to improve obesity related comorbidities and quality of life (QoL)^12^ ^,^ ^14^. The Bariatric Analysis and Reporting Outcome System (BAROS) evaluates the results of obesity treatments by analyzing three domains: weight loss, changes in co-morbidities, and QoL. Up to 3 points are allowed for each, and points are deducted for complications and reoperations. The final score classifies the results in five outcome groups (failure, fair, good, very good, and excellent), providing an objective definition of success or failure. The system was updated by Oria and Moorehead in 2009^15^. The updated BAROS includes the percentage of excess body mass index loss, new criteria for the diagnosis of diabetes, and clarifies the concept of its “improvement”.

In this study, it was aimed to evaluate the impact of LSG on patient quality of life, weight loss, and comorbidities associated with morbid obesity according to the updated BAROS criteria.

## METHODS

Eleven hundred thirty-eight adult patients were undergone to LSG by our bariatric surgery team between January 2013 and January 2016. All patients were met criteria qualifying for a bariatric surgery, i.e. BMI (body mass index) exceeding 40 kg/m^2^ or BMI exceeding 35 kg/m^2^, when diagnosed with obesity related diseases such as type 2 diabetes, hypertension, and obstructive sleep apnea. 

**FIGURE 1:**
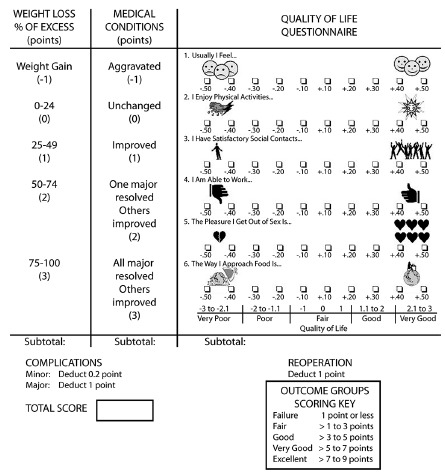
Bariatric analysis and reporting outcome system (BAROS) with Moorehead-Ardelt Quality of Life Questionnaire II scoring key

Informed consent was obtained from all individual participants included in the study. After obtaining approval from the Institutional Review Board and after appropriate permission to reproduce the updated BAROS (Figure 1) was obtained, a web-based survey including username, identity name, weight loss, the Moorehead-Ardelt Quality of Life questionnaire II (M-A QoLQ II) for pre- and postoperative periods, changes in medical conditions (diabetes, high blood pressure, sleep apnea, dyslipidemia, heart disease, arthritis, heartburn, venous leg ulcers, urinary incontinence, intertrigo), complications and re-operations was published on the Facebook group of our patients’ social committee. All responses were collected and tabulated on the Google Drive. The data from the respondents were cross-checked with hospital records for re-operations, complications, weight loss, and changes in medical conditions. The updated BAROS scores were assigned to each patient according to the scoring systems established by Oria and Moorehead. All procedures performed in studies involving human participants were in accordance with the ethical standards of the institutional and/or national research committee and with the 1964 Helsinki declaration and its later amendments or comparable ethical standards.

### Statistical analysis 

Results were expressed as mean±SD or rates. The Student’s t-test was used to compare parametric data of two groups (comparisons for preoperative and postoperative results of self-esteem and activity level scores, and mean time between surgery and questionnaire in bad- or good-resulted subgroups). The independent-samples Kruskal-Wallis test was used for the comparisons of non-parametric data as mean excess weight loss (EWL) rates or Moorhead-Ardelt II scores between five subgroups according to time between surgery and questionnaire. The bivariate analysis was used to evaluate the correlation between the updated BAROS score and EWL rate. All calculations were performed using the IBM SPSS version 22. Value of p<0.05 was considered as statistically significant.

## RESULTS

**FIGURE 2:**
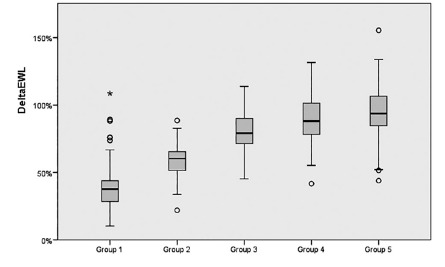
The mean EWL rates of the groups

**FIGURE 3:**
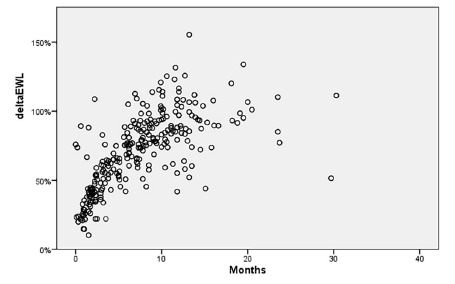
The distribution of EWL% of the patients

Number of respondants was 562 (49.4%). Seventy-one percent of respondents were female. Mean age was 34.1±8.1 years (20-56). Preoperatively, the patients had a mean weight and BMI of 129.0±20.1 kg (85-210) and 45.4±5.4 kg/m^2^ (35.1-73.8), respectively. After a mean 7.4±5.3 months follow-up (1-30), the patients achieved a mean BMI of 31.1±6.4 kg/m^2^ (18.1-6.7 kg/m^2^), respectively. When patients subdivided into five group according to time between surgery and questionnaire (quarterly for first year and others), 141 patients in group 1 (followed-up to three months); 87 patients in group 2 (followed-up three to six months), 130 patients in group 3 (followed-up six to nine months), 100 patients in group 4 (followed-up nine to twelve months), and 104 patients in group 5 (followed-up longer than twelve months). Mean EWL% was 39.9±18.1 in group 1, 59.6±12.2 in group 2, 80.8±14.9 in group 3, 89.9±18.2 in group 4, and 93.6±20.7 in group 5 (Figure 2). The mean EWL% significantly increased in subsequent groups (p<0.001), except group 5 (p=1.0). The overall EWL% was 71.3±27.1 (range, 10.2 % to 155.4 %) (Figure 3). 

Six of 1138 patients (0.5%) had leakage. All patients who had it were respondants. One closed spontaneously. Remainings were treated by endoscopic stenting. Other complications in respondants were wound infection (n=18, 3.2%), intraabdominal bleeding (n=9, 1.6%), gastroesophageal reflux (n=2, 0.4%), weight regain (n=2, 0.4%), acute mesenteric venous thrombosis (n=2, 0.4%), slipping of proximal gastric tube into posterior mediastinum (n=1, 0.2%), twisting (n=1, 0.2%), pulmonary embolus (n=1, 0.2%), intraluminal bleeding (n=1, 0.2%). Overall complication rate was 7.7%. Five sleeved stomach converted to Roux-en-Y gastric bypass because of gastroesophageal reflux, weight regain, or twisting. In a patient, it was observed that proximal part of sleeved stomach slipped into posterior mediastinum and underwent hiatus repair and gastropexy at postoperative second day. Other complications were treated medically.

The respondants reported 772 comorbidities (Table 1). Of these medical conditions, 14 (1.8%) were aggravated, 176 (22.8%) were not changed, 162 (30.0%) were improved, and 420 (54.4%) were resolved. The number of major comorbidities was 515. Of major comorbidities, 11 (2.1%) were aggravated, 124 (24.1%) were not changed, 102 (19.8%) were improved, and 278 (53.9%) were resolved. Table 1 shows the range of comorbidities.

**TABLE 1:** The range of comorbidities

Comorbidities	Aggravated (%)	Not changed (%)	Improved (%)	Resolved (%)	Total (%)
Major	11 (2.1)	124 (24.1)	102 (19.8)	278 (53.9)	515 (100)
Diabetes	2 (1.3)	39 (26.7)	34 (23.3)	71 (48.6)	146 (100)
Hypertension	1 (0.9)	31 (26.5)	22 (18.8)	63 (53.8)	117 (100)
Sleep apnea	4 (3.0)	20 (15.0)	25 (18.8)	84 (63.2)	133 (100)
Dyslipidemia	4 (3.4)	34 (28.6)	21 (17.6)	60 (50.4)	119 (100)
Minor	3 (1.2)	52 (20.2)	60 (23.3)	142 (55.3)	257 (100)
Total	14 (1.8)	176 (22.8)	162 (30.0)	420 (54.4)	772 (100)

**FIGURE 4:**
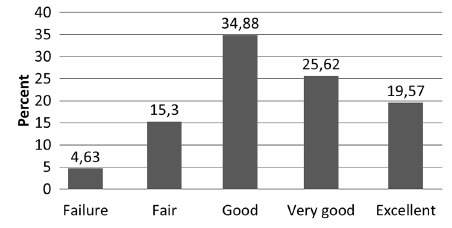
The distribution of the updated Bariatric Analysis and Reporting Outcome System - BAROS - results

Patients also reported positive self-esteem and activity level scores. The mean scores for how patients feel about themselves, enjoyment of physical activity, satisfaction with social contacts, ability to work, pleasure from sex, and their approach to food were significantly increased after LSG (Table 2). The mean updated BAROS scores were 4.0±2.4 in group 1, 4.6±2.1 in group 2, 4.6±2.1 in group 3, 5.2±2.1 in group 4, and 5.7±2.2 in group 5. It was significantly higher in group 4 and group 5 than other groups (p<0.001). The updated BAROS score was significantly correlated with EWL% in bivariate analysis (p<0.001). When these results were incorporated into the modified BAROS scoring system, 26 patients (4.6%) were classified as failures; 86 (15.3%) fair; 196 (34.9%) good; 144 (25.6%) very good, and 110 (19.6) excellent results (Figure 4). The patients were accumulated into two subgroups according to BAROS scores: bad resulted (failure or fair) and good resulted (good, very good, or excellent). Mean time between surgery and questionnaire was 5.3±5.0 months in bad resulted subgroup, while it was 8.1±5.2 months in good resulted subgroup (p<0.001). Ninety-six of 112 bad-resulted patients (85.7%) were in the first three quarterly postoperative periods. The mean updated BAROS score in patients who had complications was 4.5±1.4 (2.6-7.7) in a mean 10.5±6.2 (1.3- 20.5) months follow-up. The mean EWL% was 79.7±33.7 (33.5- 155.4) in those. The mean updated BAROS score was not significantly different than those of patients who had not complications (p<0.05). 

**TABLE 2:** Pre- and postoperative self-esteem and activity level scores

Self-esteem and activity level scores (mean±SD)	Preoperative	Postoperative	p value
Feeling about themselves	3.7±2.7	8.2±2.5	<0.05
Enjoyment of physical activity	3.4±2.9	7.9±2.7	<0.05
Satisfaction with social contacts	4.8±3.1	8.5±2.4	<0.001
Ability to work	5.5±3.2	8.5±2.5	<0.001
Pleasure from sex	4.5±3.0	7.7±2.9	<0.001
Approach to food	4.2±3.4	7.6±3.2	<0.001

## DISCUSSION

The obesity and its related diseases are massively increasing health problems in contemporary world. LSG is one of the most chosen bariatric procedures, although it is a relatively new stand-alone bariatric operation among many well-established others. The success of a bariatric procedure is assessed by considering not only the excess weight loss, but also changes in medical conditions and QoL^4^ ^,^ ^13^. Some authors use standardized tools such as Short Form-36, EuroQol Five Dimensions Questionnaire or Gastrointestinal Quality of Life Index to assess QoL after bariatric surgery. However, multiple outcome factors, such as EWL%, QoL, changes in medical conditions, and complications should be taken in consideration to assess the results of bariatric surgery. The updated BAROS is very useful for evaluating and reporting the results of obesity treatments^15^. It is obesity-specific and very simple to answer. It also evaluates reoperations in addition to above mentioned outcome factors. However, there are a few study which has a limited number of patients validating LSG by BAROS^3^ ^,^ ^5^ ^,^ ^7^ ^,^ ^10^ ^,^ ^11^. 

LSG provides acceptable percentage of weight loss and good global BAROS outcomes. Bobowicz et al.^3^ reported that mean excess weight loss (EWL) was 43.6% at 12 months in 112 patients undergone to LSG. Excellent global BAROS outcome was achieved in 13% of patients, very good in 30%, good in 34.5%, fair 9.5% and failure in 13% patients in their series. They also reported that comorbidities improved or resolved in numerous patients: arterial hypertension in 62%, diabetes mellitus in 68.3%, respectively. Similarly, D’Hondt et al.^5^ reported the mean %EWL of 83 patients was 72.3±29.3% at a median follow-up point of 49 months. The mean BAROS score was 6.5±2.1, and a ‘‘good’’ to ‘‘excellent’’ score was observed for 75 patients (90.4%). For the patients who reached the 6-year follow-up point, the mean %EWL was 55.9%±25.55%. So, they concluded that LSG is a safe and effective bariatric procedure, although a tendency for weight regain is noted after 5-years of follow-up evaluation.

On the other hand, Lemanu et al.^11^ also reported that weight loss at 5-year follow-up were modest after LSG. The mean %EWL was 40% at 5^th^ year. The mean BAROS score was 3.13 in their series including 55 patients. Recently, Felsenreich et al.^9^ presented the first complete 10-year follow-up of 53 consecutive patients who underwent LSG. They achieved a mean maximum %EWL of 71±25% at a median of 12 (12-120) months after LSG. At 10 years, a mean %EWL of 53±25% was achieved by 32 patients. Nineteen of the 53 patients (36%) were converted to Roux-en-Y gastric bypass or duodenal switch due to significant weight regain (n=11), reflux (n=6), or acute revision (n=2). Mean BAROS score was 2.4±2.2 at 10-years follow-up, classifying LSG as “fairly efficient”. 

It is well-known reality that LSG is a very effective restrictive procedure especially in short- and mid-term. It is foreseeable that a patient undergone to a restrictive procedure can not achieve long-term success without dietary calorie intake restriction or increased energy consumption by physical activity. Keren et al.^10^ reported 114 patients followed-up for 5-years after LSG. Mean EWL was >65% during initial 3-years and declined to 45.3 % in 5-years. Of the patients, 71.92% did not reach 50% EWL at 60 months. BAROS scores were 7.15 and 4.32 at 30 and 60 months, respectively. At the 5-year follow-up visit, they asked to patients whether they had significantly changed their lifestyle in the manner of nutritional habbits and physical activity. Analyzing the 32 patients with EWL >50 % in the 5-year group, 26 (81.25 %) of them had scored ≥0.5 on the two lifestyle modification questions compared with 6 (18.75 %) that scored <0.5 (p<0.001). So, they concluded that the basis for the success of LSG is knowledge and implementation of better nutritional habits and increasing physical fitness or, in other words, in significant lifestyle modification.

Although it has been a long time more than one decade through the description of sleeve gastrectomy, there is no agreement on a standart technique. Bougie size, distance from pylorus where the staple line is initiated, distance to the esophagogastric angle where the staple line is finished, and removing of fat pad are still controversial issues^2^ ^,^ ^8^. All can affect the volume of sleeved stomach and widely accepted choices vary day-by-day. Furthermore, the power of laterally traction on greater curve during vertical resection of stomach can cause a tight or loose sleeve formation leading to satisfactory %EWL or not. It is conceivable that the patients with the longest follow-up have been operated by the less experienced surgeons on LSG. As far as we know, there is no study evaluating how results were affected if a surgeon changed own preferences on the aforementioned issues. We prefer a 39-Fr bogie size, starting to resection 2 to 4 cm from pylorus, removing of fat pad, finishing the resection at the esophagogastric angle, and creating a thight sleeve to reduce residual gastric volume as well as possible.

In the present study, we reported the results of 562 patients who undergone LSG. The mean EWL increased until 1^st^ postoperative year and then patients keeped their weights during postoperative 2^nd^ year. The rates of improving or resolving comorbidities were 71.9% for diabetes, 72.6% for arterial hypertension, 82.0% for obstructive sleep apnea, and 68.0% for dyslipidemia. The mean updated BAROS scores of patients significantly increased at 4^th^ postoperative quarter, and it also stretched during 2^nd^ year. The EWL% of patients who were followed up at least 12 months was significantly higher than previous studies, as well as improvement rate in comorbidities. The proportion of patients whose their updated BAROS scoring classified as failure or fair was only 19.9 %. And the majority of those (85.7%) was in the first three quarterly postoperative periods. It can be expected that rate will be further reduced when one-year follow-up is completed. The high success rate in this study can be attributed to the preferred surgical technique to reduce residual gastric volume or the efficiency of patient support group on social media. Because nearly a half of patients has participated in survey. It is likely that they encourage each other to continue the lifestyle modification on the Facebook group. 

The present study may be criticized because of the limited follow-up. However, the number of patients who had been followed-up for longer than one year in this study is comparable to previous papers. As far as we know, it is the largest series with respect to QoL following LSG. The preferred surgical technique was particularly emphasized in this study unlike previous reports on QoL after LSG. It may be effective on the results of procedure. Another censurable issue is that data received by web-based questionnaire. The evaluated objective criteria in the updated BAROS such as re-operations, complications, weight loss, and changes in medical conditions were cross-checked with hospital database. The M-A QoLQ II, which is part of BAROS, is a self-assessment questionnaire. So, the sending out method of a mail questionnaire has no effect on reliabilities of the answers. But some subgroups of patients, such as younger people, may be more likely to use internet technologies and e-mail. The major concern is that different response rates of subgroups may lead to a bias in the study^16^. There is a lack of evidence about the use of internet technologies to influence response rates in clinical trials. The present study is not first clinical trial delivering the M-A QoLQ II via web-based. Janik et al.^9^ sent the M-A QoLQ II via e-mail to patients. The response rate was 19% in patients had undergone to bariatric surgery. The rate of response in our study is significantly higher than that of Janik et al.^9^. We prefer to use Facebook’s messenger to communicate with patients. The higher response rate can be attributed to the fact that the preferred way to delivery the questionnaire is the same as the preferred way to communicate with patients. It can be assumed that the method for delivering questionnaire had not great impact on results, because the high response rate in the study reduced the bias possibility.

## CONCLUSION

LSG is a highly effective bariatric procedure in the manner of weight control, improvement in comorbidities and increasing of QoL in short- and mid-term. Its success rate in long-term may be related to surgical technique, as well as lifestyle modification. 
